# Industrial Implementation of Aluminum Trihydrate-Fiber Composition for Fire Resistance and Mechanical Properties in Glass-Fiber-Reinforced Polymer Roofs

**DOI:** 10.3390/polym14071273

**Published:** 2022-03-22

**Authors:** Mohammad Zainudin, Kuncoro Diharjo, Mujtahid Kaavessina, Djoko Setyanto, Ubaidillah Ubaidillah

**Affiliations:** 1 Mechanical Engineering Department, Engineering Faculty, Universitas Sebelas Maret, Surakarta 57126, Indonesia; mzaind2003@yahoo.com (M.Z.); ubaidillah_ft@staff.uns.ac.id (U.U.); 2 Chemical Engineering Department, Engineering Faculty, Universitas Sebelas Maret, Surakarta 57126, Indonesia; mkaavessina@staff.uns.ac.id; 3 Mechanical Engineering Department, Atma Jaya Catholic University of Indonesia, Jakarta 12930, Indonesia; djoko.setyanto@atmajaya.ac.id

**Keywords:** composite, aluminium trihydrate (ATH), unsaturated polyester resin, mechanical properties, fire-resistant

## Abstract

It is difficult to obtain suitable fire resistance and mechanical properties for glass-fiber-reinforced polymer (GFRP) roof material in industrial applications. Although some efforts to improve the fire resistance properties of GFRP have been carried out, in practice this sometimes degrades the mechanical properties. Therefore, the base materials, such as filler and reinforcing fiber, must be appropriately combined to simultaneously improve both fire resistance and mechanical properties. The present study examines improvements in GFRP roof material by investigating the effect of aluminium trihydrate (ATH) as a filler and the combination of a chopped strand mat (CSM) with woven roving (WR) and stitched mat (STM) fibers as the reinforcement in a composite GFRP roof structure. The roof samples were prepared following industrial machine standards using the specified materials. The mechanical properties of GFRP were evaluated using tensile, flexural and impact tests, following ASTM D638, ASTM D790 and ASTM D256 standards, respectively. The fire properties were examined through fire tests following the ASTM D635 standard. The results show that the GFRP roof composed of CSM/WR fibers had a 40% higher tensile strength (103.5 MPa) compared with the GFRP roof without CSM fibers (73.8 MPa). The flexural strength of the GFRP roof with CSM/WR fibers was also 57% higher than the roof without fibers, with a ratio of 315.61 MPa to 201 MPa. With the use of CSM/WR fibers, the fire resistance also increased by 23%, resulting in a ratio of 4.31 mm/min to 5.32 mm/min. These results demonstrate that the combination of CSM/WR fibers as a reinforcement would be an excellent option for producing an improved GFRP roof with better industrial properties, especially when producing improved GFRP roofs using a continuous lamination machine.

## 1. Introduction

Glass-fiber-reinforced polymer (GFRP) consists of high-strength fibers within a polymer resin, namely, unsaturated polyester resins (UP resins). Compared with pure UP resins, the utilization of glass fiber is typically preferred to enhance the strength, stiffness, corrosion resistance, fatigue resistance, or strength-to-weight ratio of the polymer. In industry, GFRP is generally utilized in airplanes, electronic components, automotive applications, railways, and sporting goods. The widespread use of GFRP composites results from the ease with which their mechanical properties can be modified as desired. However, due to the effects of tropical climatic variables (ultraviolet, humidity, temperature fluctuations, rain, etc.), the mechanical properties of GFRP are prone to deterioration over time [1,2]. Additionally, GFRP composites tend to have a combustible matrix because unsaturated polyester resins are polymers with chains containing considerable amounts of carbon and hydrogen [3]. This problem is often solved by adding fillers into the matrix to increase the polymer fire resistance, some of which include aluminium trihydrate (ATH), boric acid (AB), and sodium silicate (Na_2_SiO_3_).

Several previous studies have revealed the effect of fire retardants on GFRP composites, both on their mechanical and physical properties [4,5,6,7,8,9]. The type of fire retardant used, as well as its load, significantly affects the mechanical properties. In general, adding a fire retardant reduces mechanical properties [4]. The addition of ATH to GFRP as a filler has been observed to weaken tensile strength, which decreases due to poorer bonding between the filler powder, matrix, and the glass fiber used [5,6,7]. It is also argued that the addition of ATH is not recommended for applications that require heavy loading due to the associated decrease in mechanical properties. In contrast, F. Perez-Salinas et al. [8] revealed that the addition of 9% ATH does not significantly impact the tensile strength and modulus of elasticity of the composites compared with the addition of magnesium hydroxide. However, the addition of ATH filler can enhance fire resistance by decreasing the burning rate [9].

Moreover, various fire-retardant fillers, such as ATH, DeBDE, Sb_2_O_3_, and TCP, have been tested to produce compounds with the desired properties. ATH has been demonstrated to particularly improve mechanical properties; however, even loading up to 60% does not confer any fire-retardant properties [10]. Additionally, Kaleg et al. [11] found that aluminium trihydrate (Al(OH)_3_) filler was able to increase the initial temperature of the decomposition of UP resin, thereby reducing the burning rate of GFRP composites.

Other studies have revealed that reinforcing fibers might affect the properties of GFRP composites [12,13,14,15]. The addition of reinforcing materials such as glass or carbon fiber can increase the strength and modulus of rPP/clay composite, thereby making possible its application as a structural component in vehicle bodies [16]. Abburi Lakshman Kumar et al. [17] observed that the direction of fiber reinforcement impacted the mechanical properties and machinability of GFRP composites. A fiber direction of ±45 exhibited improved tensile and flexural strength compared with a 0/90 combination. Bushra Rashid et al. [18] reported that the mechanical properties of the composites improved as the number of layers of glass fiber increased. Composites with Woven/UP resin showed better effect strength, whereas CSM/UP resin composites showed preferable flexural strength over Woven/UP resin. Notwithstanding, the mixture composite Woven/CSM/UP resin had by far the best mechanical properties [17,18].

Given the benefits, the need for industrially manufactured GFRP composite roofing with improved mechanical properties and fire resistance is significantly growing among users. Therefore, the creation of GFRP composite roofs with improved properties has become necessary. Recent developments in GFRP composite roofing with CSM fiber reinforcement have included adding sand material to the matrix to increase fire resistance [19,20,21]. However, these products are still only made on a laboratory scale using hand lay-ups, rather than a roof-making machine due to the constraints when setting process parameters in the machine. This is a shortcoming because the market demand for GFRP composite roofing has increased.

On the other hand, no composite roofs have been manufactured that use fire-retardant ATH as a filler and reinforcing fiber from a combination of CSM, Woven, or even Stitchmat (STM) fibers. One example of GFRP composite roofs currently available is that produced by PT Intec Persada, Indonesia [22]. This GFRP roof is made of a composite of UP resin matrix and 30% ATH filler, reinforced with CSM E-glass fiber. Based on the specifications, the roof has a tensile strength of 73.8 MPa, a flexural strength of 201 MPa, a burning rate of 5 mm/min, and its properties could be still further improved [23]. Therefore, this study investigates the use of ATH as a filler with a combination layer configuration of E-glass fibers such as CSM, WR, and STM with the aim of developing a GRFP composite roof with better fire resistance and mechanical properties than those currently available on the market.

## 2. Materials and Methods

This study addresses the need to improve the performance of GRFP composite roofs made at a composite roofing factory at PT. Intec Persada, Tangerang, Indonesia [22]. The matrix was composed of unsaturated polyester resin (UP resin) and aluminium trihydrate (ATH) (orthophthalic UP resin, PT. Justus Kimiaraya, Jakarta, Indonesia, YUKALAC^®^, LP1QEX, Lot #: A1400613 AG). Up to 1% of the methyl ethyl ketone peroxide (MEKP) of the total resin volume was used to initiate the matrix hardening process. In addition, ATH delivered by PT. Kawaguchi Kimia Indonesia under the code BW153 was used as a fire-retardant filler. All the materials were used directly without any treatment.

The reinforcing fiber used was an E-glass CSM of Jushi, Tongxiang, China with a density of 450 g/m^2^ (CSM 450) and 300 g/m^2^ (CSM 300), E-glass WR with a density of 200 g/m^2^ (WR 200), and an E-glass stitch mat with a density of 450 g/m^2^ (STM 450).

Two- or three-ply composite plates were fabricated by employing a hand lay-up method. E-glass fibers within CSM 300, CSM 450, WR 200, and STM 450 were impregnated with the matrix. MEKP was used as a catalyst, which was first added to the resins to start the polymerization process. A gel coat was applied as a decorative and protective surface on the outside layer. The layer configurations of the three samples are tabulated in Table 1.

**Figure 1:**
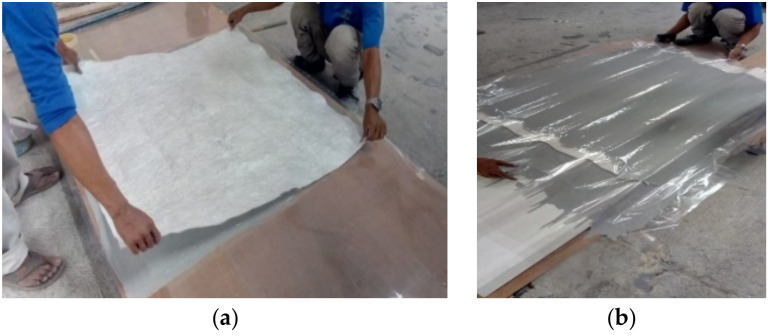
(**a**,**b**) Layering and molding of a GFRP composite roof.

The amount of ATH used in Sample 1 was 30% and increased to 45% for Samples 2 and 3. Each sheet was designed with a thickness of up to 1.5 mm. Then, to match the actual roof, each composite sheet was molded by pressing between molds in the form of a roof sheet. Then, a curing process at room temperature was performed, as shown in Figure 1a,b.

**Figure 2:**
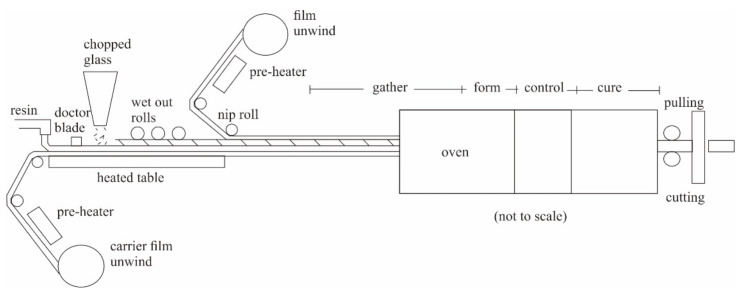
A schematic of continues laminating process.

Figure 2 shows a schematic of the continuous lamination process [24]. Two sheets of plastic film were used to wrap the composite layer. The reinforcing fiber used was CSM, roving, or STM. The reinforcing fiber with resin mixed with the initiator was synthesized in bulk. Curing was carried out at a temperature of 50–140 °C in an oven at a 60–120 kg/h production rate. UP resin liquid mixed with the initiator was dropped on the bottom plastic film; then, chopped strand mat or pieces of roving were placed on top. Wet-out rolls removed air bubbles. Next, the plastic film was coated on the top side. Finally, the entire layer was pulled into the oven by the pulling system and heated with a specified temperature distribution: experience has shown that good lamination results are obtained at a resin gel temperature of 80–90 °C and a peak exothermic temperature of 150–165 °C. The flat or corrugated sheets produced in the oven were cured into solids and then cut to the desired length.

**Figure 3:**
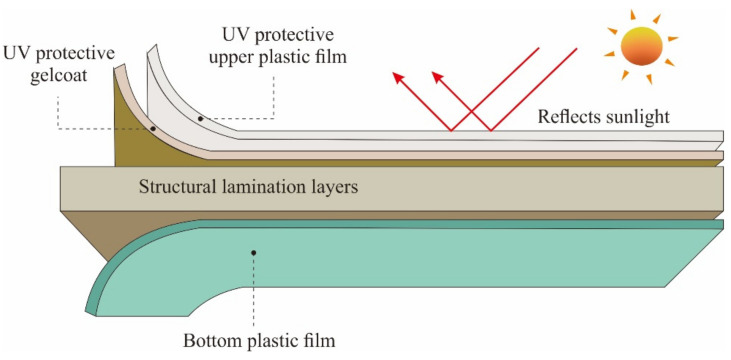
The layer structure of the GRFP roof.

Our GFRP composite roof had two UV protection layers: an anti-UV film layer and an anti-UV gel-coat layer. Double-layer protection as shown in Figure 3 maintains the quality of the composite roof, making it more durable against weather, UV rays, and humidity. 

**Figure 4:**
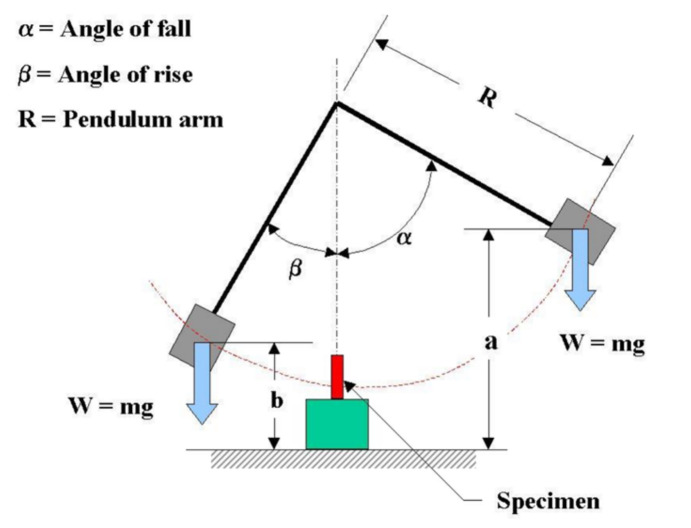
Izod type Impact test according to ASTM D256.

The flexural properties of the GFRP roof were ascertained from the three-point bending test following the ASTM D790 standard. The test samples were obtained by cutting from the flat section of the sheets for each type of GFRP composite. There were seven samples of each composite being tested with the thickness of each sample measured to be 50.8 mm long by 12.7 mm wide using a UTM JTM-UTS510 series testing machine at room temperature with a test speed of 1.4 mm/min. The tests for tensile properties were performed on each sample according to ASTM D638. The tensile test was conducted on five samples of each composite tested, and the dimensions of each test specimen were 115 mm long, 25 mm gauge length and 6 mm width as required by ASTM D638 Type IV. The tensile test was conducted using a UTM Shimadzu Type AG-250kN Plus with a test speed of 2 mm/min. An Izod type impact test was performed according to ASTM D256. There were five test pieces of each sample with the dimensions 63.5 × 12.7 × 3.2 mm. The test was performed using a standard-sized cantilever beam clamped vertically into the tester stop with a 2 mm V-notch at a 45-degree angle just above the clamp. Based on the test configuration shown in Figure 4, the impact energy was calculated by the formula E = W.R (cos α − cos β) in Joule.

**Figure 5:**
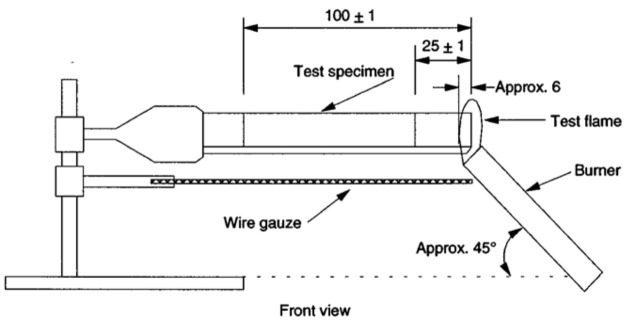
Burn test equipment according to ASTM D635.

The rate of burning was in reference to the ASTM D635 standard as illustrated in Figure 5, the fireside propagation on flammable materials in a horizontal position was assessed on five test pieces of each sample. SEM observations were employed to determine the morphology of the specimen fracture surface, especially on the bond between the filler and the matrix. An SEM observation using a ZEISS EVO 10 apparatus at 15 kV also aimed to examine the filler dispersion of the matrix. We observed the likelihood of filler agglomeration within the matrix. The thermogravimetric analysis (TGA) test was conducted to measure the change in mass (decrease or increase in mass) and the rate of change of mass as a function of temperature. The TGA test was conducted by measuring the mass of the sample in a pan using a precision balance placed in the furnace. In measuring the mass degradation of polymer composite specimens, the test environment used controlled atmospheric air. The heating rate parameter was set at 10 °C/min and the test temperature range was from room temperature to 600 °C. Finally, a differential scanning calorimetry (DSC) test was also carried out which was used to determine the endothermic and exothermic reactions of the specimen.

## 3. Result and Discussion

**Figure 6:**
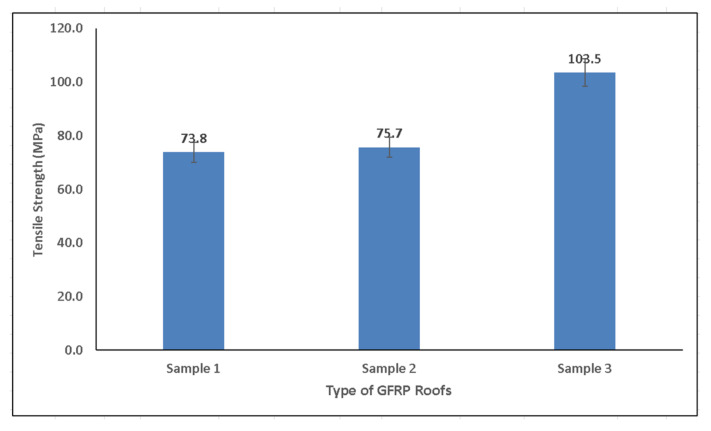
Tensile strength of GFRP composite roofs.

Table 2 exhibits the test result of all samples in this research. The bar graph in Figure 6 shows that the GFRP composite roof of Sample 3 had a higher tensile strength than Samples 2 or 1. The tensile strength of the composite roof of Sample 3 reached 103.5 Mpa, which was higher than the tensile strength of Sample 2 (75.7 Mpa) and Sample 1 (73.8 Mpa). This indicates that the glass fiber inserted into the matrix as reinforcement increased the tensile strength. Glass fibers are typically used to improve strength, stiffness, and fatigue resistance, or enhance the strength-to-weight ratio over pure UP resin [11,21]. As illustrated in the data above, the tensile strength was not subject to the type of composite matrix reinforcement—CSM, STM, or WR—but was strongly influenced by the composite density. A similar situation was reported in previous studies, showing that the number of reinforcing fiber layers does affect the mechanical strength properties of GFRP composites [17,18,21]. In this study, Sample 3 had a greater density than Samples 1 and 2.

**Figure 7:**
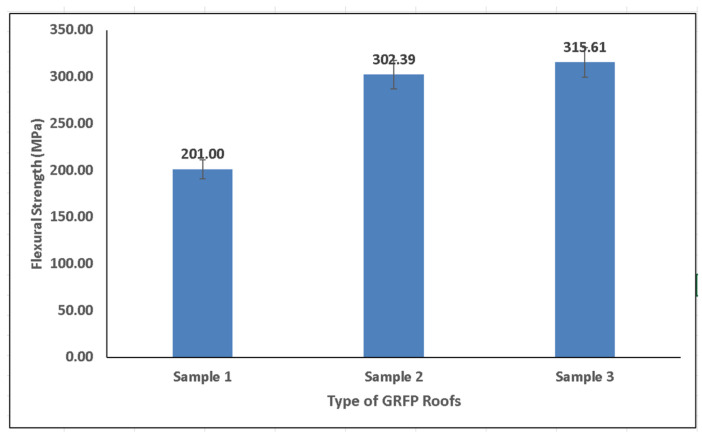
Flexural strength of GFRP composite roofs.

Figure 7 shows that the flexural strength of the GFRP material for Sample 3 was almost identical to that of Sample 2. However, it was higher than Sample 1. The flexural strength of Sample 3 reached 315.61 Mpa, which was not substantially different from that of the GFRP in Sample 2 (302.39 Mpa); however, the flexural strength of Sample 3 was higher than that of Sample 1 (201 Mpa). Therefore, it can be deduced that different layer combinations (CSM/STM or CSM/WR) can provide space for movement, leading to a higher flexural strength vis à vis the strength of the same layer combination (CSM/CSM) in Samples 2 and 3.

**Figure 8:**
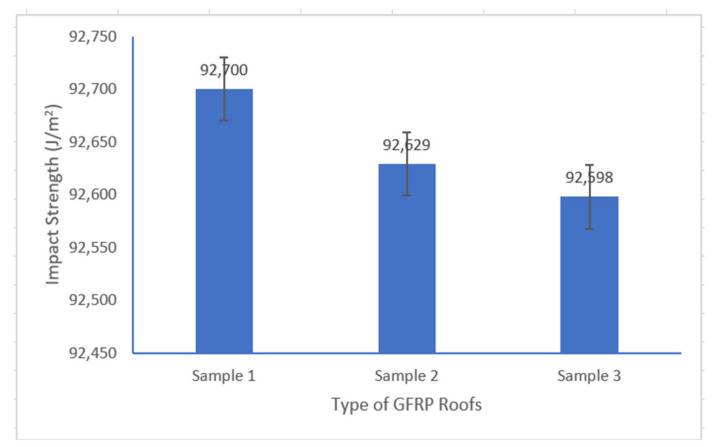
Impact strength of GFRP composite roofs.

Impact testing was carried out to determine how much energy was absorbed by the GFRP composite roof before fracture. As shown in Figure 8, the GFRP composite roof of Sample 3 had increased impact strength compared with Sample 2 and Sample 1, exhibiting a ratio of 92,598:92,629.5:92,700 J/m^2^.

**Figure 9:**
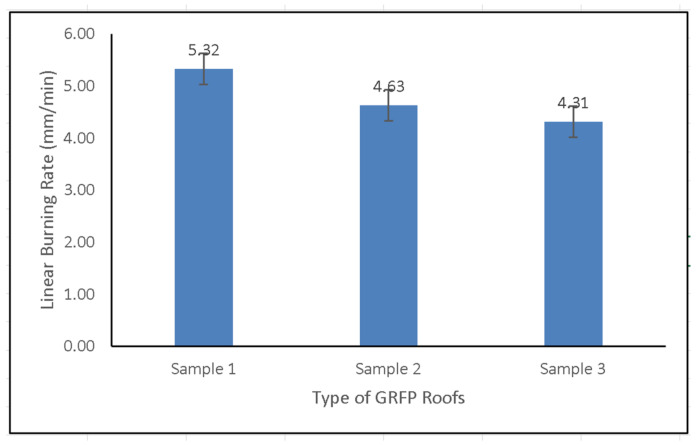
Linear burning rate of GFRP composite roofs.

A burn test was conducted to determine the burning rate of the samples. In this test, all composites were tested following the ASTM D635 standard and included in the HB class (horizontal burning), where the burn rate was still below 75 mm/min. It was discovered that the linear burn rate of the GFRP composite roof in Sample 1 reached 5.32 mm/min, whereas Samples 2 and 3 had linear burn rates of 4.63 mm/min and 4.31 mm/min, respectively, as shown in Figure 9. The findings demonstrate that the layer type (CSM, WR, or STM) does not substantially affect the burning rate, although altering the layer density does increase fire resistance.

Composites with STM fibers can improve the mechanical performance by up to 20% compared with CSM fiber types. The reasons are that STM fibers are parallel non-crimp fibers that can withstand strain immediately after loading, and their higher density can be incorporated into laminates compared with CSM. The fibers behave more as a unidirectional layer [25]. It can be argued that the more significant the volume fraction of the reinforcing fiber, the higher the strength and stiffness values of the resulting composite [26]. In the current experiment, the density of the sample with WR reinforcement was greater than that of CSM or STM reinforcement, which is consistent with the rule of mixture. However, there is a maximum volume fraction for the reinforcing fiber, which is determined by the length and orientation of the selected reinforcing fiber [26].

The decrease in the combustion rate of Sample 1 was caused by heat absorption. The thermal energy was reduced in the composite due to the presence of H_2_O. In other words, a reduction in thermal energy causes the burning rate of the composite to decrease [27]. Umberto Berardi et al. [28] reported that as the mass of ATH is increased, the mass-loss rate per unit area (MLRPUA) diminishes, and the burn rate decreases. Adding aluminium hydroxide as a fire retardant will decrease the burn rate. It breaks down at around 180 °C (356 °F) due to exposure to too much heat and releases water vapor.

The additional density resulting from the reinforcement material can increase fire resistance even at a minimal composition [29]. Additionally, the use of glass fiber in the phenolic matrix could result in high tensile strength and good fire resistance, as per the UL94 standard [30]. Here, the dehydration of ATH and cleavage of the ester bond result in the first significant mass loss, forming volatile products such as H_2_O, dicyclopentadiene, benzaldehyde, and dimethyl phthalate [31].

Adding ATH to UP resin composites increases both the limiting oxygen index (LOI) and ATH load, improving fire resistance [32]. Increased fire resistance indicates that ATH may prolong the time to ignition (TTI) and reduce the heat release rate (HRR) [33]. The particle size of ATH affects the fire resistance of the UP resin; thus, equal mass ratios of submicron fillers and nanofillers provide the most straightforward fire resistance [34]. High ATH loads also result in an increase in the modulus of elasticity and a decrease in fracture elongation, increasing the stiffness and brittleness of the composite. The modulus of elasticity of the UP resin’s 40% ATH load is 2.41 Gpa, 29.57% higher than the modulus of elasticity of the pure UP resin [35]. Increasing the ATH load of a GFRP composite tends to reduce the bending strength. The addition of ATH particles to GFRP composites can result in the non-uniform distribution of ATH particles and increased strain concentration in the fracture process [27]. Therefore, the addition of ATH may reduce the strength and make the GFRP more brittle.

**Figure 10:**
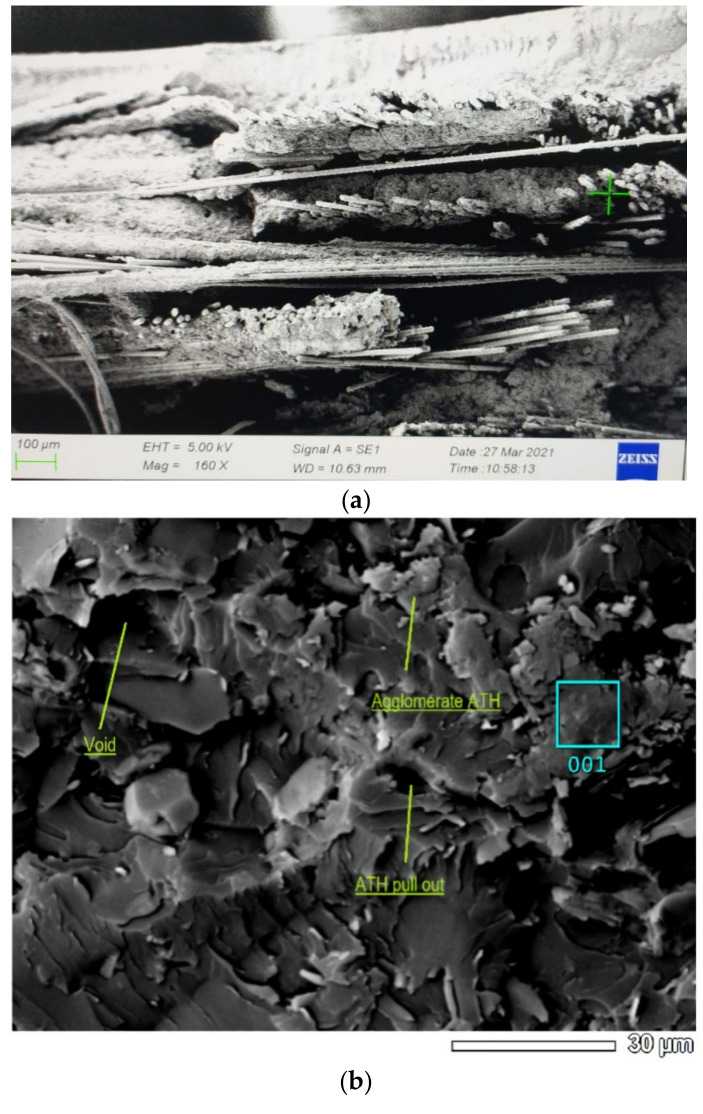
Micrograph result: (**a**) SEM of ATH distribution in UP resin; (**b**) SEM of stitch mat GFRP composite roof fracture.

The SEM image in Figure 10a shows the arrangement of the stitched mat fibers in the cross-sectional direction, where the fiber-like portion of the seam reinforces the woven fibers that form it. A stitched mat reinforcing fiber structure has more fibers than woven or CSM reinforcement. The stitched mat fibers have a coiled fiber in a seam, allowing for better bonding between the fibers than CSM. In addition, the higher density of WR reinforcing fibers compared with reinforcements with STM or CSM fibers indicates more mechanical strength, which is also consistent with the mixture principle [26].

This study shows that the increase in ATH content in GFRP composites up to 45% tends to form agglomerations. As shown in Figure 10b, agglomeration produces an inhomogeneous distribution of particles, weakening the interaction between the filler and the matrix [36]. In addition, ATH agglomeration triggers stress concentration in the area around the particles, resulting in decreases in the mechanical properties of the composite [24]. In summary, ATH is a rigid particle that is difficult to deform; therefore, an increase in ATH content in the UP resin can cause an increase in the modulus of elasticity of the composite.

**Figure 11:**
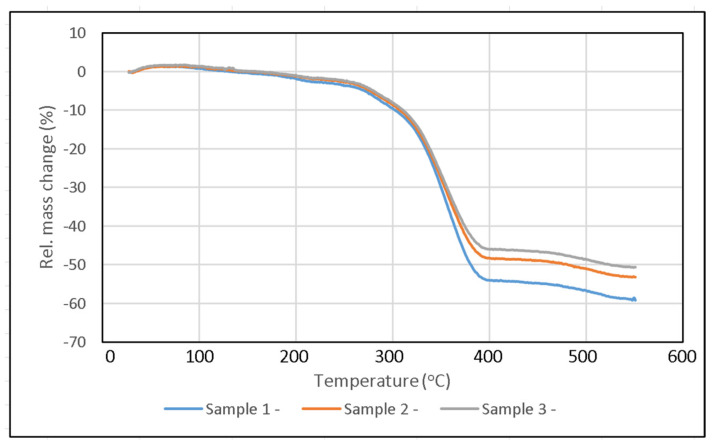
TGA curve result.

The ATH content in UP resin showed a decrease in mass degradation due to the release of moisture. The tested specimens exhibited a loss of moisture up to a temperature of 250 °C [37,38]. As shown in the TGA curve in Figure 11, at a temperature of 250 °C, Sample 1 showed a mass degradation of 3.7%, whereas Sample 2 and Sample 3 exhibited 2.9% and 2.15%, respectively. These figures indicate that Sample 1 experienced the highest decomposition when the moisture release process occurred. This is consistent with the SEM images which show that ATH particles tend to form agglomerations in the UP resin. The decomposition temperature at 50% mass degradation of Sample 1 was 380 °C. Meanwhile, the decomposition temperature at 50% mass degradation of Sample 2 and Sample 3 increased by 100 °C and 144 °C, respectively, compared with Sample 1. The decomposition of UP resin in each sample occurred at a temperature of 280 °C to 400 °C. At temperatures above 400 °C, the graph in Figure 11 shows a relatively flat line. This indicates that decomposition did not continue even though the specimen temperatures increased. The final residue of these samples contained alumina which has a melting temperature of 2054 °C, much higher than the melting temperature of UP resin [32,33].

From the TGA test results curve in Figure 11, it can be seen that in Sample 1 there was a mass reduction of about 0.05% at a temperature of 130 °C, whereas about 0.3% and 1% mass reductions occurred in Samples 2 and 3, respectively. This mass reduction occurred due to evaporation of moisture and other materials on the surface of the composite [39]. At temperatures above 280 °C, a very significant mass reduction began to occur up to a temperature of 400 °C. The gradual decrease in mass above 400 °C could be due to the slow oxidation of stable carbon produced during decomposition [39].

**Figure 12:**
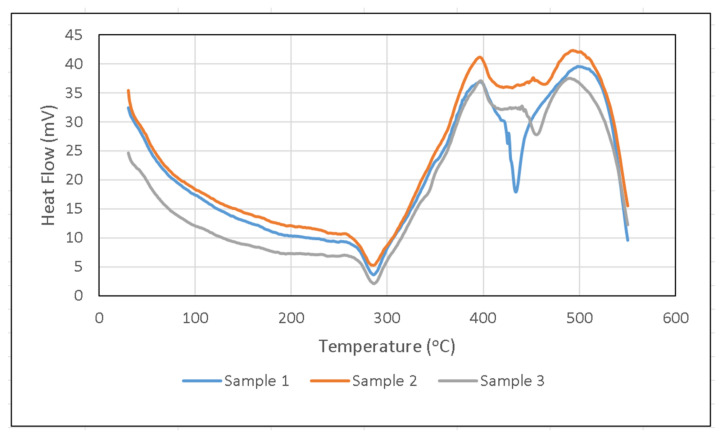
DSC curve result.

As shown in the DSC test result curve in Figure 12, in Sample 1, from the beginning of the test, the curve showed a slow endothermic reaction which is indicated by a near horizontal sloping curve. At temperatures less than 200 °C, the absorbed heat resulted in moisture evaporation and volatilization due to the initial decomposition process [39]. The curve began to rise at 280 °C and an exothermic peak occurs at 400 °C, due to the thermal oxidation of the volatiles with oxygen which generates heat (exothermic). The occurrence of these peaks was due to the large energy required during thermal decomposition to break the crosslinks of polystyrene, which was then followed by evaporation of polystyrene and volatiles [33]. The temperature continued to increase so that endothermic peaks appeared at 370 °C and 380 °C (close to 400 °C). This solid phase oxidation came from the slow oxidation of stable carbon produced during decomposition [40].

In Sample 2 and Sample 3, before the temperature of 300 °C, an endothermic peak was seen at 280 °C. This endothermic reaction describes the start of the thermal decomposition of the composite which produces volatiles. As these volatiles break down into smaller fractions they oxidize more readily [41]. The exothermic peak appears at 400 °C. This exothermic reaction describes the thermal oxidation of volatiles with oxygen which produces heat. ATH filler will prevent the oxidation that occurs following the endothermic reaction during filler decomposition. Filler decomposition produced water vapor which played a role in reducing the combustion rate. The water vapor escapes the fire and dissolves the concentration of combustible gases from the polymer matrix. This process limits oxygen access on the composite surface [37]. As the ATH filler began to decompose at 200 ºC, the endothermic reaction of ATH and water vapor during decomposition absorbed the heat of combustion and dissolved combustible gases. In addition, ceramic alumina (Al_2_O_3_), which functions as an insulating layer against heat, will be formed [42]. The exothermic peak that occurred in the three samples with the filler was around 400 °C, which means it was in the main decomposition area, where the greatest mass degradation occurred in the TGA test. This illustrated the severance of the polystyrene crosslink, which was then followed by the evaporation of the polystyrene and volatiles [33].

## 4. Conclusions

The GFRP composite roof using the combination of CSM/WR fibers had a 40% higher tensile strength than the GFRP roof with CSM/CSM fibers as reinforcement, with a respective ratio of 103.5:73.8 MPa. The flexural strength of the former was 57% higher than that of the latter. Furthermore, for the three types of the GFRP roofs investigated there was little difference in the impact strengths, which were 92,700 J/m^2^, 92,629 J/m^2^, and 92,598 J/m^2^ for Samples 1, 2, and 3, respectively. The fire resistance, as estimated by the linear burn rate of the investigated GFRP roofs, differed by 23% between the roof with the combination of CSM/WR fibers and that with CSM/CSM fibers as reinforcement, which corresponds to a ratio of 4.31:5.32 mm/min. This investigation offers promising applications in producing new, improved GFRP roofs with better mechanical characteristics and fire resistance properties. Combining CSM/WR fibers as reinforcement is recommended to produce new and improved GFRP roofs using a continuous lamination machine.

## Figures and Tables

**Table 1:** Layer configurations of the composite.

Sample	Layer Configuration
**Sample 1**	CSM 300—CSM 450 (existing GFRP composite roof)
**Sample 2**	CSM 300—STM 450
**Sample 3**	CSM 300—WR 200—CSM 300

**Table 2:** Data sample test result.

Type of Sample	Tensile Test				Flexural Test				Izod Impact Test				Burn Test			
	No of Specimen	Force_(max)_ (N)	Strength (Mpa)	AVR (Mpa)	No of Specimen	Force_(max)_ (N)	Strength (Mpa)	AVR (Mpa)	No of Specimen	Energy (J)	Strength (J/m^2^)	AVR (J/m^2^)	No of Specimen	Burning Time (min)	Linear Burning Rate (mm/min)	AVR (mm/min)
Sample 1	1	652.1346	72.5400	73.8	1	78.3	205.5686	201.00	1	3.635602	90,213.44047	92,700.4	1	12.80	5.55	5.32
	2	707.0479	75.3702		2	77.1	234.2267		2	3.636279	90,230.24489		2	12.75	5.25	
	3	682.6799	73.2175		3	77.1	177.9065		3	3.636279	92,762.21605		3	13.07	4.90	
	4	702.5248	74.4200		4	76.9	200.3033		4	3.636279	93,237.91972		4	13.03	5.37	
	5	701.3226	73.4523		5	78.8	178.9882		5	3.636279	97,058.02400		5	12.98	5.16	
					6	79.4	206.8151						6	12.55	5.74	
					7	77.4	203.2057						7	13.02	5.30	
Sample 2	1	902.4540	86.4627	75.7	1	131.2	344.4521	302.39	1	3.639876	93,330.16021	92,690.0	1	9.85	5.69	4.63
	2	758.2110	66.2482		2	127.8	292.5736		2	3.639876	89,630.04797		2	13.72	4.23	
	3	597.0000	59.3605		3	125.7	372.9214		3	3.639332	90,306.00898		3	11.35	3.70	
	4	857.2340	85.9245		4	125.3	326.3720		4	3.639876	93,330.16021		4	13.25	4.75	
	5	832.9150	80.5738		5	115.1	237.1458		5	3.639876	96,548.44159		5	10.88	5.33	
					6	109.3	286.9559						6	12.38	4.28	
					7	110.2	256.3183						7	13.38	4.41	
Sample 3	1	1060.6100	124.9240	103.5	1	124.1	286.3580	315.61	1	3.622783	92,891.86853	92,597.7	1	10.78	5.19	4.31
	2	906.2690	96.9634		2	128.2	259.9767		2	3.622783	92,182.77030		2	9.35	4.92	
	3	839.3920	102.4820		3	124.2	320.9794		3	3.621751	92,156.51497		3	12.52	3.91	
	4	924.2300	95.1794		4	136.7	356.0658		4	3.621751	92,865.41124		4	8.50	4.82	
	5	908.6390	98.0283		5	131.4	300.8151		5	3.621751	92,891.86853		5	10.02	4.39	
					6	130.6	340.1770						6	12.05	3.49	
					7	132.4	344.8655						7	13.67	3.44	

## Data Availability

Not applicable.
